# The electric field changes the anomalous properties of the Mercedes Benz water model

**DOI:** 10.1039/d2cp05670d

**Published:** 2023-01-11

**Authors:** Tomaz Urbic

**Affiliations:** a Faculty of Chemistry and Chemical Technology, University of Ljubljana, Askerceva 5 SI-1000 Slovenia tomaz.urbic@fkkt.uni-lj.si

## Abstract

The influence of a homogeneous constant electric field on water properties was assessed. We used a simple two-dimensional statistical mechanical model called the Mercedes-Benz (MB) model of water in the study. The MB water molecules are two-dimensional disks with Gaussian arms that mimic the formation of hydrogen bonds. The model is modified with added charges for interaction with the electric field. The influence of the strength of the electric field on the water's properties was studied using Monte Carlo simulations. The structure and thermodynamics of the water were determined as a function of the strength of the electric field. We observed that the properties and phase transitions of the water in the low strength electric field does not change. In contrast, the high strength electric field shifts boiling and melting points as well as the position of the density maxima. After further increasing the strength of the electric field the density anomaly disappears.

## Abstract 2

The influence of a homogeneous constant electric field on water properties was assessed.

## Graphical abstract

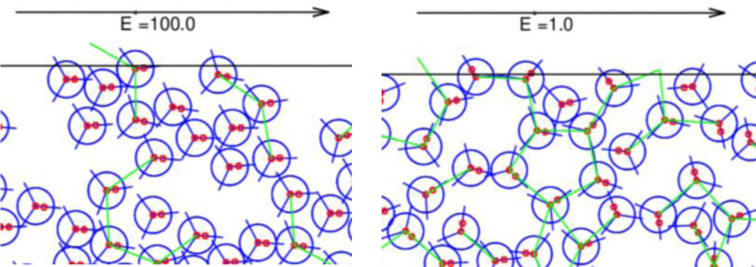

## I Introduction

Water is an essential part of all the processes on Earth and the most unusual compound.^1^ When compared to other materials, the exceptional anomalous properties put it in a class by itself. When we talk about the anomalous properties, we mean that the behaviour of water is entirely different from what is observed in other liquids.^2,3^ The most known anomalous properties are a density maximum; a lower density of the solid phase in comparison to the liquid phase; almost constant heat capacity in the liquid phase; a negative expansion coefficient; high surface tension, and viscosity. The reason for these anomalies is the formation of hydrogen bonds (HB) among water molecules. This bonding looks like as if water molecules attach themselves to other water molecules or other substances. Each water molecule can form four HBs; two by donating its hydrogen atoms and two by accepting hydrogen atoms. This process forms a local tetrahedral arrangement of the water molecules which is the reason for the counter-intuitive anomalies. The strength and directionality of the hydrogen bonds control liquid waters’ thermodynamic and dynamic behaviour. To understand the behaviour and properties of the water and aqueous solutions it is therefore crucial to understand hydrogen bonding and its molecular background.

Because of its small size, polarity and high dielectric constant, water is an excellent solvent.^4,5^ It is a perfect solvent for polar and ionic compounds and salts. It also has unique hydration properties for biological macromolecules like nucleic acids and proteins. The water hydration for these molecules determines the three-dimensional structures and functions in solution.^6–15^ Water molecules play a dominant role in a variety of charge transfer determined phenomena.^16^

An external electric field changes the distribution of the electron density. Its application induces the polarizability of electrons, atoms, and dipoles, resulting in eventual reorientation of molecules along the applied electric field. As such, the electric field is one of the important factors that is able to modify the properties of the water.^17–19^ The electric field can occur because of ions, dissolved in the water, or it can be applied externally, for example, generated by cracks in crystals. In aqueous solutions ions reorient neighbouring water molecules and form hydration shells; moreover, some ions can hydrolyse water molecules. In case where the interaction energy between a water dipole and an external electric field of a given charge distribution, *e.g.* an ion, may be considered small with respect to thermal fluctuations or hydrogen bond energies, the dielectric response is linear. Experimental^17,18^ studies report an increase of temperature of the liquid–solid transition under the influence of external electric fields. In a relatively weak field, the authors ascribed freezing to two factors: the confinement and the ice formed without dipole reorientation along the field. Strong fields are found to increase the freezing temperature. The change of the phase diagram was also discovered for bulk water in an external electric field,^19^ where the electric field can shift transitions between ice phases and alter the freezing temperature. Strong electric fields also have complex effects on ice formation and dissociation. Druchok and Holovko studied the changes of the structural and dynamic properties of water molecules exposed to electric fields, external ones or generated by ions.^20^ They performed a set of molecular dynamics simulations for a series of systems modelling aqueous electrolyte solutions and pure water. The systems presenting pure water were exposed to external fields of a different strength. The external fields within the studied range can slow down water molecules and even crystallize the system. Recently, the behaviour of liquid water under the effect of an electric field was studied by *ab initio* molecular-dynamics.^21–24^ It was determined that the hydrogen-bond length and the molecular orientation are significantly modified at low-to-moderate field intensities. Electric fields beyond a threshold of about 0.35 × 10^10^ V m^−1^ are able to dissociate molecules and sustain an ionic current *via* a series of correlated proton jumps.

Despite extensive theoretical and experimental studies, the reason for water properties that come from its molecular structure still remains poorly understood. A large number of models of varying complexities to model water's extraordinary properties have been developed and analysed; for reviews, see, *e.g.* Ref. 6, 7, and 25–28. There have been two main approaches to modelling liquids. The first one was performing computer simulations of atomically detailed models. The second one was to build simplified models and to capture as many properties of the water and aqueous solutions as possible by such models.

One of the simplest water models is the so-called Mercedes-Benz (MB) model^29^ which was originally proposed by Ben-Naim in 1971.^30,31^ It is a 2-dimensional toy model where each water molecule is represented with a disk that interacts with other disks that represent water molecules through a Lennard-Jones (LJ) interaction and an orientation-dependent hydrogen bonding interaction with three radial arms arranged as in the MB logo. When the two MB particles are at a correct distance and have the two arms collinearly aligned, the HB is formed between these particles. Simplified models are interesting because they give the insights that are not obtainable from all-atom computer simulations. Simpler models are more flexible in providing insights and illuminating concepts, and they also do not require vast computer resources. These simple models can be used as a sandbox, where theoretical methods can be developed and studied. Our interest in using the MB model is that it serves as one of the simplest models for the orientationally dependent liquid, thus it can serve as a testing ground for developing analytical theories that might ultimately be useful for more realistic models. Another advantage of the MB model, compared to the more realistic water models, is that the underlying physical principles can be more readily explored and visualized in two dimensions. The *NpT* Monte Carlo simulations have shown that the MB model has many similarities to real water such as: the density anomaly, the minimum in the isothermal compressibility as a function of temperature, the large heat capacity, as well as the experimental trends for the thermodynamic properties of solvation of nonpolar solutes,^29,32–34^ and cold denaturation of proteins.^35^ The MB model was also extensively studied by analytical methods such as thermodynamic perturbation theory and integral equation theory.^36–42^

In this work, we studied the interaction of bulk MB water molecules with the homogeneous constant electric field of different strengths. We used a modified version of the MB model which has previously been used by Hribar-Lee *et al.*^43^ This type of model only allows us to study the interplay between electric field and hydrogen bonds. This paper is organized in the following way. The next section introduces the model used in this study, followed by the details of Monte Carlo simulations. It continues in a section where the results are presented and discussed. The paper concludes with a summary in the final section.

## II The model

**Fig. 1:**
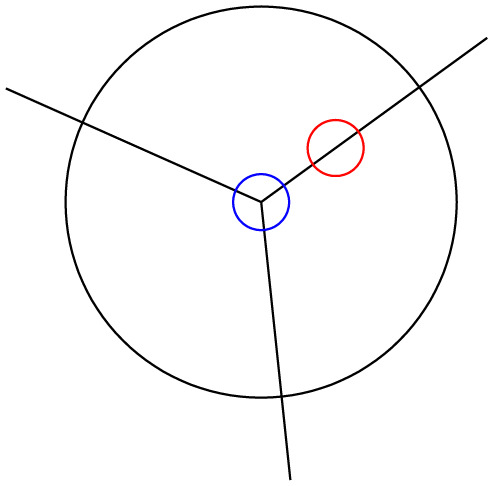
The MB model of water. For the interaction in the electric field, the red circle represents a positive charge, and the blue represents a negative charge.

In the MB model, each water molecule is represented as a two-dimensional Lennard-Jones disk with three attached arms that mimic the formation of the hydrogen bonds (see Fig. 1). An angle between each of the two arms is 120°.^29–31^ Interaction is the sum of the Lennard-Jones (LJ) term and hydrogen-bonding (HB) term which depends on the distance between molecules and their orientation and can be written as:

1 *U*(*X⃑*_*i*_, *X⃑*_*j*_) = *U*_LJ_(*r*_*ij*_) + *U*_HB_(*X⃑*_*i*_, *X⃑*_*j*_)

*X⃑*_*i*_ represents a vector of position and orientation of the *i*-th particle, and *r*_*ij*_ is the distance between *i*-th and *j*-th particles. The LJ term is calculated in a standard way2
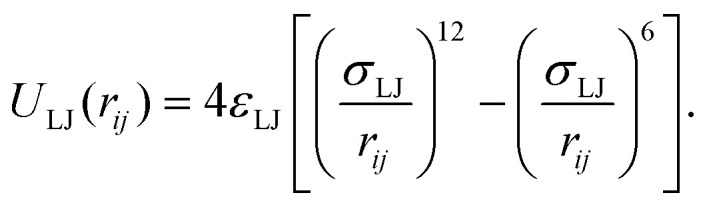
*ε*_LJ_ and *σ*_LJ_ are the depth and the contact distance of the LJ potential. The HB term is a sum of all interactions *U*^*kl*^_HB_ between the arms *k* and *l* of molecules *i* and *j*, respectively3
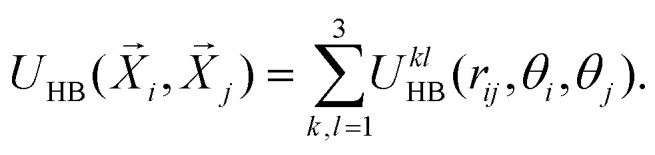
*θ*_*i*_ and *θ*_*j*_ are the orientations of the *i*-th and *j*-th particles. The HB interaction is a product of Gaussian functions depending on the orientation of each molecule and their distance

4 *U*^*kl*^_HB_ (*r*_*ij*_, *θ*_*i*_, *θ*_*j*_) = *ε*_HB_*G*(*r*_*ij*_ − *r*_HB_)*G*(*i⃑*_*k*_*u⃑*_*ij*_ − 1)*G*(*j⃑*_*l*_*u⃑*_*ij*_ + 1)

5
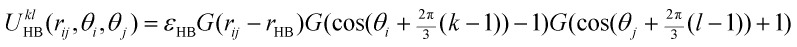
where *G*(*x*) is an unnormalized Gaussian function:6
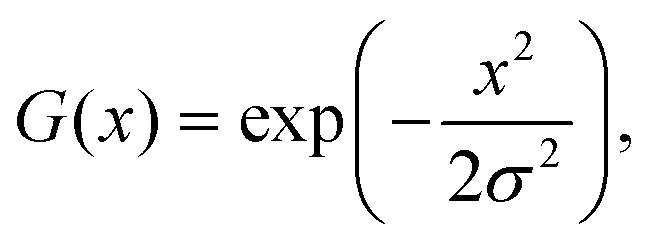
*ε*_HB_ is the HB energy and *r*_HB_ is a HB distance. *u⃑*_*ij*_ is the unit vector in the direction of *r⃑*_*ij*_ and *i⃑*_*k*_ is the unit vector of the *k*-th arm of the *i*-th particle. When the distance between two molecules is *r*_HB_, and the orientation of these two molecules is positioned so that their interacting arms are parallel and pointing towards each other's centres, the interaction between molecules is the strongest. We used the same measuring units as in previous studies: energies were expressed in |*ε*_HB_| and lengths in *r*_HB_.^29^ Thus the energy parameter for hydrogen-bonds, *ε*_HB_, was −1, and the hydrogen-bond length was 1. The same width parameter *σ* = 0.085 was used for both the distance and the angle deviation of a hydrogen bond. Parameters of the LJ potential were set to: *ε*_LJ_ = 0.1|*ε*_HB_| and *σ*_LJ_ = 0.7*r*_HB_. The model we used for particles to interact with the electric field was the modified MB model previously used by Hribar-Lee *et al.*^43^ which includes an electrostatic dipole. A single negative charge is put at the center of each water molecule. A single positive charge was placed onto one of the H-bonding arms, at a distance 0.165*r*_HB_ from the center (see Fig. 1). The other two H-bonding arms were not charged. This dipole was added only for interaction between water molecules and the electric field, and water–water interaction was the same as described before.

## III Monte Carlo simulations

Monte Carlo (MC) simulations with the Metropolis algorithm were performed in order to determine the properties of the MB model in different strengths of constant homogeneous electric field. The MC simulations were carried out in *NpT* and *NVT* ensembles. Periodic boundary conditions and minimum image convention were used to mimic the macroscopic system. 100–400 MB particles were present in the system at all times. This number of particles in 2D is equivalent to 1000–8000 particles in 3D. The initial positions of particles were randomly chosen in a way where there was no overlap between molecules. A molecule was randomly chosen in each MC step in order to be translated or rotated. On average, each cycle consisted of one rotational and translational attempt per particle. In *NpT*, there was also an attempt to change the volume of the system once per cycle. First, the system was allowed to equilibrate for a minimum of 30 000–1 00 000 cycles, which depended on the temperature of the system. After the system was equilibrated, the sampling was performed in a 20–100 series, each consisting of minimum 30000 cycles. Phase thermodynamic quantities were calculated as statistical averages during the sampling.^45,46^

## VI Results and discussion

All results are presented in reduced units. HB energy parameter *ε*_HB_ was used to normalize temperature and excess internal enthalpy 
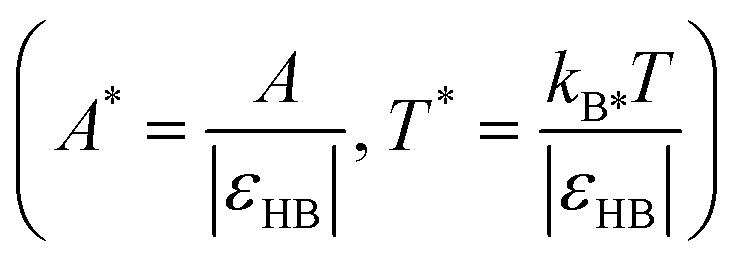
, and distances are normalized with the characteristic length of the hydrogen bond *r*_HB_
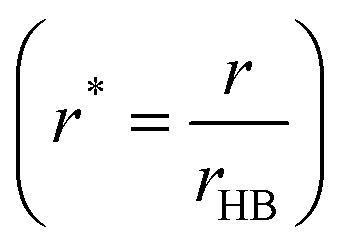
. The strength of electric field is normalized as 
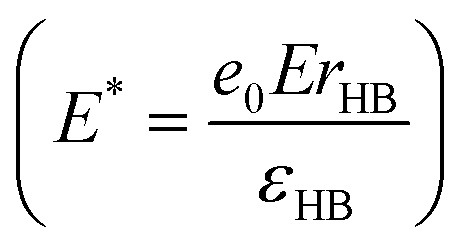
. The value E* = 100 is approximately *E* = 7 × 10^10^ V m^−1^ depending on the experimental value of *r*_HB_ and *ε*_HB_.

**Fig. 2:**
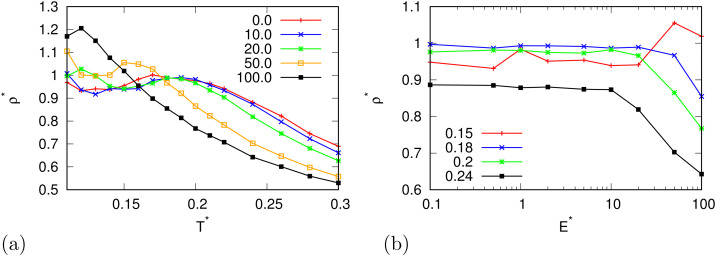
The density as a function of (a) temperature for different electric field strengths at pressure p* = 0.19 and (b) of the electric field strength.

**Fig. 3:**
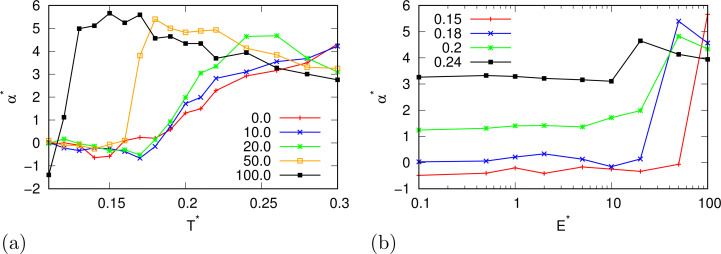
The thermal expansion coefficient as a function of (a) temperature for different electric field strengths at pressure p* = 0.19 and (b) of the electric field strength.

**Fig. 4:**
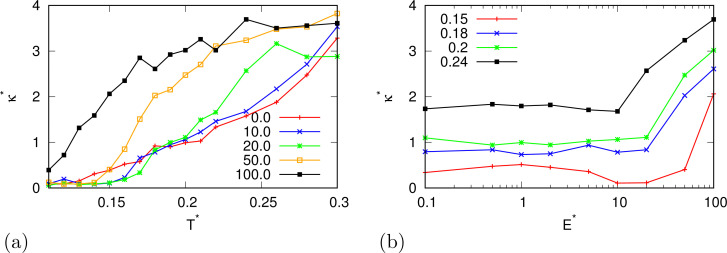
The isothermal compressibility as a function of (a) temperature for different electric field strengths at pressure p* = 0.19 and (b) of the electric field strength.

**Fig. 5:**
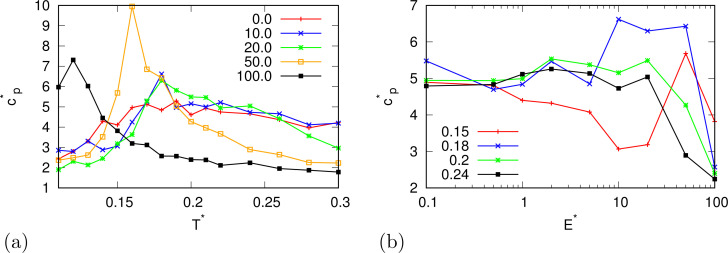
The heat capacity at constant pressure as a function of (a) temperature for different electric field strengths at pressure p* = 0.19 and (b) of the electric field strength.

**Fig. 6:**
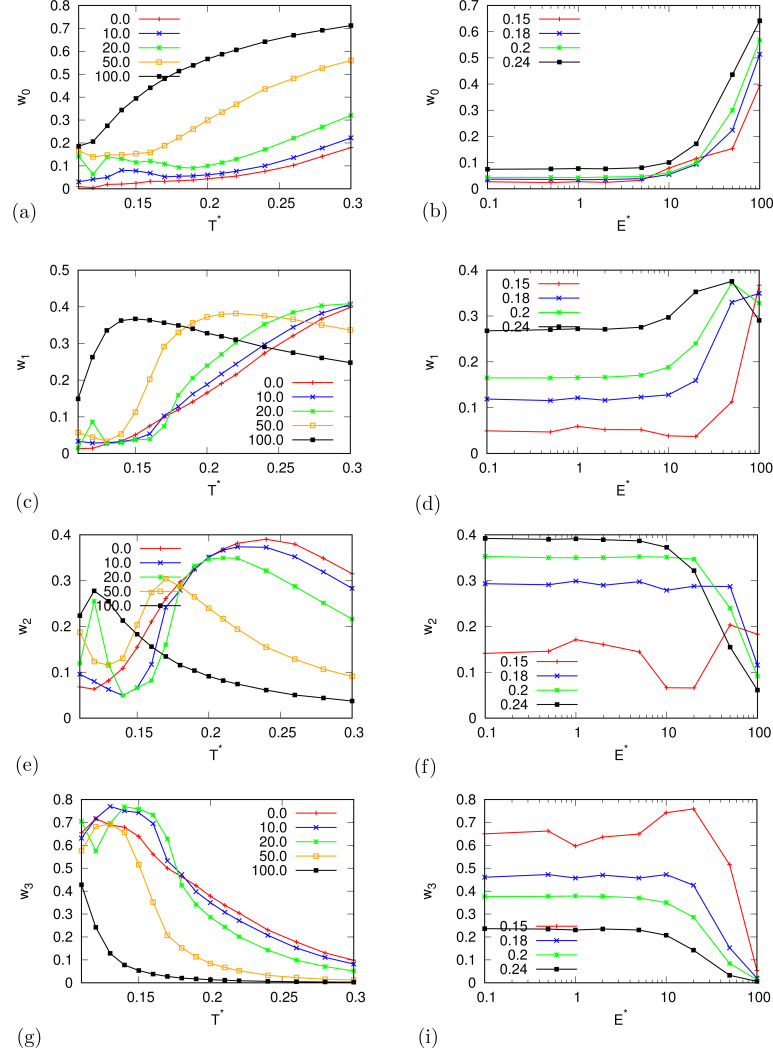
The ratios of differently bonded water molecules as a function of temperature for different electric field strengths at pressure p* = 0.19 and of the electric field strength (a and b) for the ratio of non-bonded water molecules, (c and d) ratio of the MB molecules with one HB, (e and f) with two HBs, and (g and h) with three HBs.

**Fig. 7:**
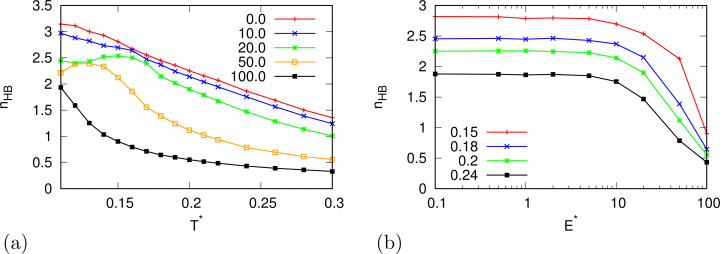
The average number of hydrogen bonds as a function of (a) temperature for different electric field strengths at pressure p* = 0.19 and (b) of the electric field strength.

**Fig. 8:**
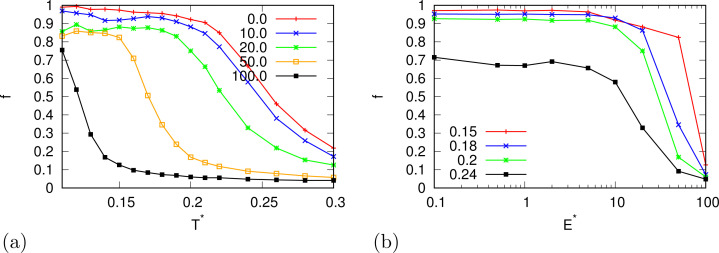
The ratio of MB water molecules in the biggest cluster as a function of (a) temperature for different electric field strengths at pressure p* = 0.19 and (b) of the electric field strength.

**Fig. 9:**
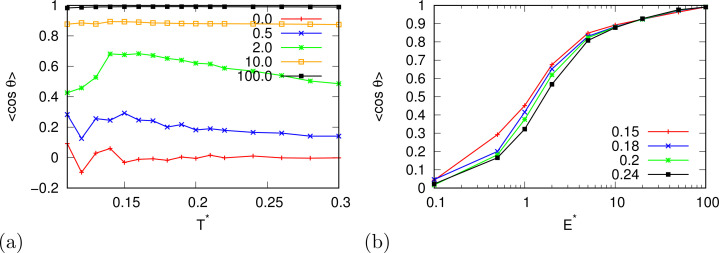
The averaged cosine of the angle of water molecules with respect to the electric field as a function of (a) temperature for different electric field strengths at pressure p* = 0.19 and (b) of the electric field strength.

**Fig. 10:**
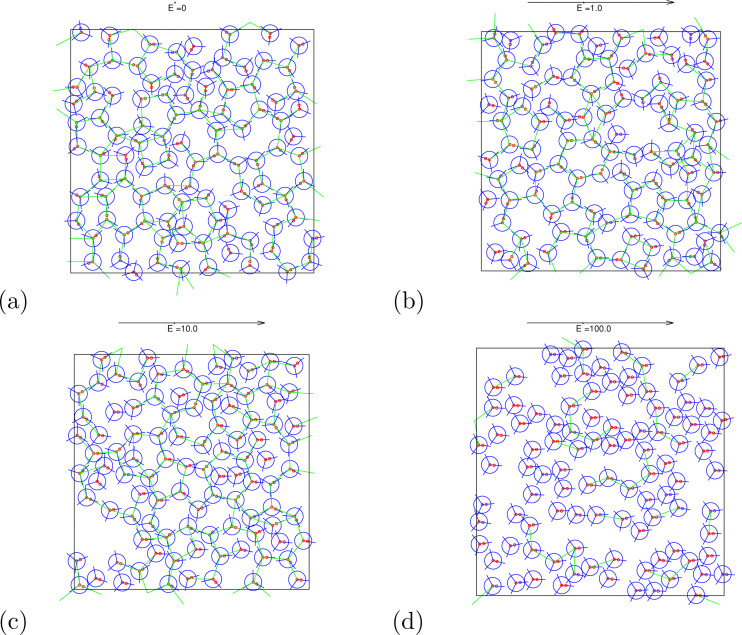
Snapshots of the system for different electric field strengths at pressure p* = 0.19 and temperature T* = 0.18. The green lines connect MB molecules that form HBs. Red marks represent charges for interaction with the electric field.

**Fig. 11:**
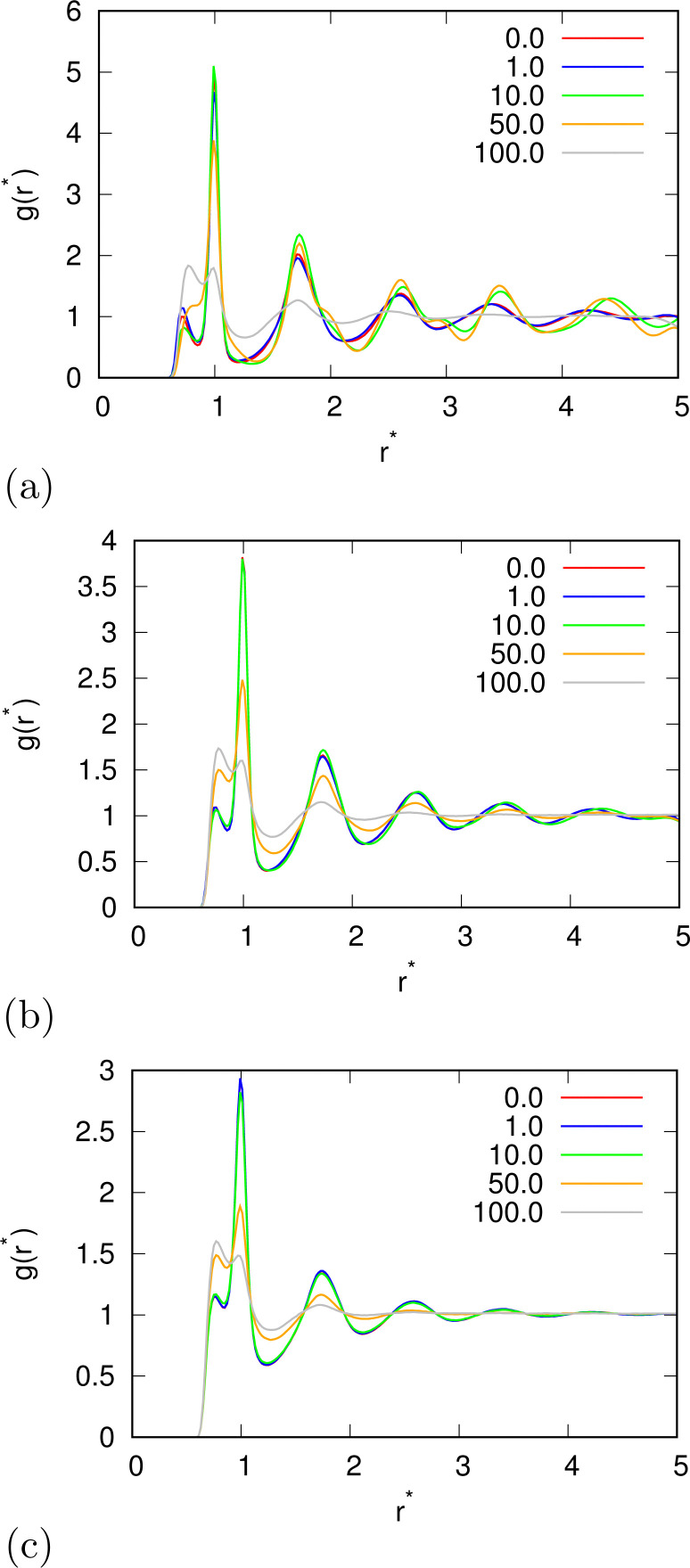
A pair correlation function between water molecules for (a) T* = 0.15; (b) T* = 0.18; and T* = 0.24; for different electric field strengths.

**Fig. 12:**
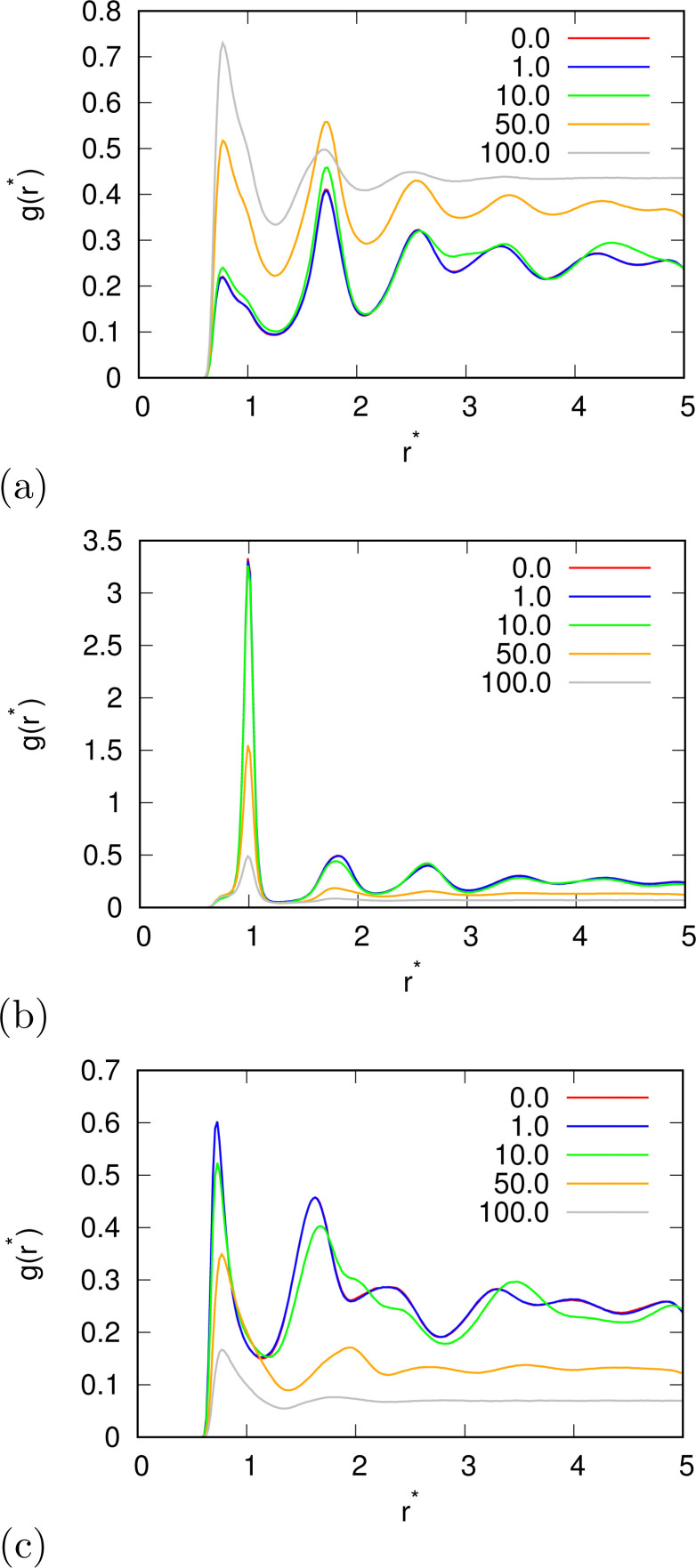
A pair correlation function between differently oriented water molecules (a) −π/6 < *θ*_1_, *θ*_2_ < π/6; (b) −π/6 < *θ*_1_ < π/6, 5π/6 < *θ*_2_ < 7π/6; and (c) 5π/6 < *θ*_1_ < 7π/6, −π/6 < *θ*_2_ < π/6.

**Fig. 13:**
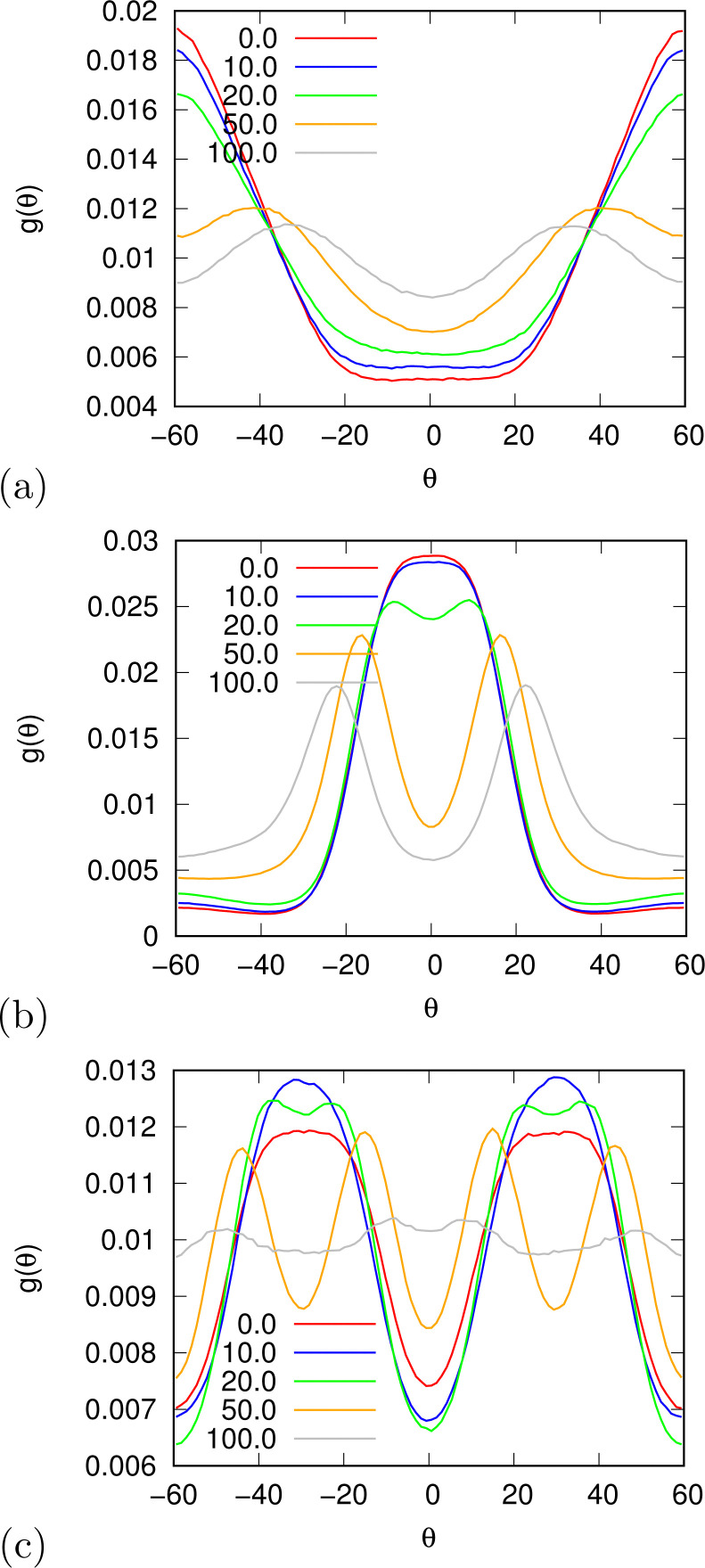
The distribution of water molecules with respect to orientation at a distance (a) r* = 0.7, (b) r* = 1.0 and r* = 1.7. Angle 0 means that the water's hydrogen bonding arm is pointing toward the center of other molecules.

**Fig. 14:**
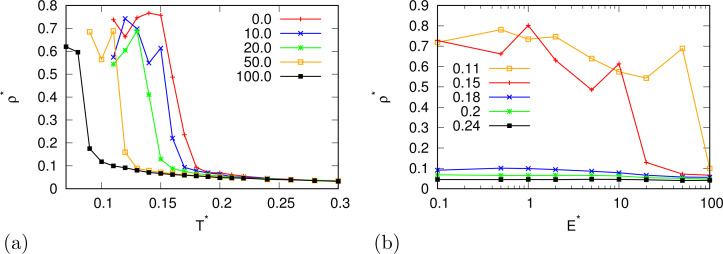
The density as a function of (a) temperature for different electric field strengths at a lower pressure p* = 0.01 and (b) of the electric field strength.

In the beginning we checked how the thermodynamic properties of the MB model change with the presence of the electric field. We have made calculations for the pressure p* 00.19. This is the pressure where previous MB simulations were reported^29^ and where the MB water exhibits density anomaly. We have plotted temperature dependence for different electric fields and electric field dependence for different temperatures of density (Fig. 2), thermal expansion (Fig. 3), isothermal compressibility (Fig. 4) and heat capacity (Fig. 5). The density has a maximum that is well pronounced at around T* = 0.18 for electric fields of up to around E* = 15, after that the position moves to lower temperatures. The same can also be seen for the thermal expansion coefficient. The coefficient is negative left of the maxima for all the electric fields. We can see that the isothermal compressibility is high for higher temperatures where we work with a liquid form, and it goes to almost 0 at low temperatures where we have a solid form. The position where it reaches almost 0 moves to lower temperatures, which indicates that when the intensity of the electric field is increased, the melting temperature decreases. We can deduct the same conclusion for the temperature dependence of heat capacity at constant pressure. The collected data indicate that increasing the electric field lowers the heat capacity for the range of temperatures where we have liquid. For the value E* = 100 electric field the heat capacity is about half of the value compared to where no field is present for temperatures higher than T* > 0.18. We also observed an interesting shape of the curve. In stronger electric fields the peak appears close to the melting temperatures. We believe that the reason for the electric field to only have this effect in stronger electric fields is the fact that at strengths 15.0 and higher the interaction energy of the MB water molecule with the electric field is higher than the average hydrogen bond energy per molecule. We continued our research by analysing the hydrogen bond network. We checked the temperature dependence for different electric fields and the electric field dependence for different temperatures of the ratio of MB water molecules where it had none, one, two, and three hydrogen bonds formed (Fig. 6), for the average number of hydrogen bonds formed per MB water molecule (Fig. 7), and for the ratio of MB water molecules in the biggest cluster (Fig. 8). We can see from the diagrams that the electric field destroys the hydrogen bond network. The ratio of MB molecules with no HB increases with the increase of the electric field for all the temperatures. We observed an increased ratio of the triple bonded MB molecules for electric fields around E* = 15, at the same time we noticed a decrease for once and twice bonded molecules for temperatures around T* = 0.15. We can claim that the HB network is enhanced in this region, even though the average number of formed HBs per molecule does not increase. An average number of HBs per molecule is lower at all temperatures for all electric fields in comparison to when there is no applied field. At the lowest temperatures, where there's no applied field, we can observe an average number higher than three because in glass state molecules two hydrogen bonds per arm can be formed. We have plotted the ratio of MB water molecules belonging to clusters that are connected by hydrogen bonds in Fig. 8. With no applied field at a temperature T* = 0.26 50% of water molecules are part of the big cluster. For the electric field E* = 20 this temperature decreases to 0.22, for the field E* = 50 to 0.17 and for the electric field E* = 100 to 0.125. We can deduct from these data that the electric field moves as a percolation curve at lower temperatures. We continued by calculating how the orientation of MB water molecules changes with respect to an electric field. We have calculated the average of cosine of the angle of the HB arm with charge with respect to the electric field. The temperature dependence for different electric fields and electric field dependence for different temperatures of this quantity which is proportional to polarization due to dipole alignment is plotted in Fig. 9. At the small electric fields, the response is linear, while at higher electric fields, it reaches a plateau since all dipoles are already aligned with an electric field as reported for the TIP4P/2005 water model.^19^ In snapshots presented in Fig. 10 we can see that the higher electric field aligns the dipoles and lowers the number of hydrogen bonds between MB particles. The electric field also destroys hexagonal structures present in MB water. In Fig. 11 we have plotted pair correlation functions for different electric field strengths. We can observe two different regimes. One is for temperatures that are higher than the temperature of the density maxima T* = 0.18. In this regime the electric field melts hydrogen bonds and increases LJ contacts. This can be seen in heights of the peaks at 0.7 (LJ contact increases meaning more LJ contacts because HB melted) and at 1.0 (HB contact decreases due to melting of HBs). The electric field also changes long range correlations. In higher electric field strength, structures decay faster toward 1 and MB molecules behave more like LJ kind of liquid. In the second regime for temperatures lower than T* = 0.18 small fields enhance HBs, which leads to higher HB peaks and longer range of osculations in correlation functions. A further increase of the field, E* = 100, for example, melts HBs like in the other regime. In Fig. 12 we split correlation function into three contributions depending on the orientation of both water molecules. We have plotted a correlation function when both water molecules are pointing in the same direction (a), opposite direction pointing toward each other (b) and away from each other (c). An increase of the electric field favours orientation in point (a) where all dipoles are aligned in the same direction. We can also see how the HB network is being melted with an increase of the field. In Fig. 13, we have drawn the orientation of water molecules at different distances from the central water molecule. These figures lead to the same conclusions as the two other figures before. We have also made calculations for much lower pressure p* = 0.01. At this pressure value, we have the gas–liquid phase transition for this model^44^ at a temperature T* = 0.16. We noticed here that a higher electric field lowers the boiling temperature (Fig. 14).

## V Conclusions

We have studied the electric field effect on the anomalous properties and phase behaviour of the 2D MB water model with the help of Monte Carlo simulations. An electric field of a low strength (E* < 15.0) does not affect the anomalous properties of water and the position of phase transitions. When a low strength electric field is applied, the hydrogen bonds are dominating in the phase space. In contrast, for higher strength electric fields the position of density maxima and boiling and melting points shift to lower temperatures. We should also stress out that the current MB model does not take into account induced dipole moment, nor does it permit dissociation of water molecules.

## Conflicts of interest

There are no conflicts to declare.

## Supplementary Material
